# *hTERT* gene methylation in circulating DNA, tumor, and surrounding tissue in breast cancer: a prospective study

**DOI:** 10.1590/1516-3180.2023.0140.R1.04032024

**Published:** 2024-05-10

**Authors:** Luiz Fernando de Queiroz, Marcelo Soares da Mota e Silva, Heitor Siffert Pereira de Souza, Siane Lopes Bittencourt Rosas, Maria da Glória da Costa Carvalho

**Affiliations:** I MD, PhD. Molecular Biologist, Department of Pathology, Universidade Federal do Rio de Janeiro (UFRJ), Rio de Janeiro (RJ), Brazil.; II MD, PhD. Molecular Biologist, Department of Pathology, Hospital Universitário Clementino Fraga Filho, Universidade Federal do Rio de Janeiro (UFRJ), Rio de Janeiro (RJ), Brazil.; III MD, PhD. Physician and Full Professor, Department of Internal Medicine, School of Medicine, Universidade Federal do Rio de Janeiro (UFRJ), Rio de Janeiro (RJ), Brazil.; IV MD, PhD. Molecular Biologist, Department of Internal Medicine, School of Medicine, Universidade Federal do Rio de Janeiro (UFRJ), Rio de Janeiro (RJ), Brazil.; V MD, PhD. Physician and Associate Professor, Department of Pathology, Universidade Federal do Rio de Janeiro (UFRJ), Rio de Janeiro (RJ), Brazil.

**Keywords:** Cell-free nucleic acids, Telomerase, Methylation, Breast neoplasms, Liquid biopsy, Telomeres, Breast cancer, Liquid biopsies

## Abstract

**BACKGROUND:**

The human telomerase reverse transcriptase (hTERT) enzyme, encoded by the *hTERT* gene, synthesizes protective telomeric sequences on chromosomes and plays a fundamental role in cancer formation. Methylation of the *hTERT* gene has an upregulatory effect, increasing hTERT enzyme synthesis and allowing continuous tumor cell division.

**OBJECTIVE:**

In a group of patients with breast cancer, we aimed to analyze the methylation status of *hTERT* in the tumor, surrounding tissue, and circulating free deoxyribonucleic acid (cfDNA) of blood collected on the day of mastectomy and then approximately one year later.

**DESIGN AND SETTING:**

A prospective study was conducted at a university hospital in Rio de Janeiro, Brazil.

**METHODS:**

Samples were collected from 15 women with breast cancer on the day of mastectomy and approximately one year postoperatively. cfDNA was analyzed by sodium bisulfite conversion, followed by polymerase chain reaction, electrophoresis, and silver nitrate staining.

**RESULTS:**

Methylation of *hTERT* was detected in the tumors and surrounding tissues of all 15 patients. Five patients displayed *hTERT* methylation in the cfDNA from the blood of the first collection. Of the ten patients who returned for the second collection, three showed methylation. Two patients with methylation in the first collection did not display methylation in the second collection. One patient with no methylation in the first collection displayed methylation in the second collection, and one patient had a diminished level of methylation in the second collection.

**CONCLUSION:**

Only one-third of patients displayed methylation in their cfDNA, which may be related to the success of chemotherapy.

## INTRODUCTION

Telomeres are repetitive deoxyribonucleic acid (DNA) sequences found at the ends of chromosomes that protect the chromosomes from degradation and ensure their stability. In each round of normal cell division, telomeres shorten, leading to cellular senescence and, on critical shortening, eventual cell death.^1,2^ Human telomerase reverse transcriptase(hTERT) is an enzyme that synthesizes telomeric DNA sequences.^3^ The hTERT enzyme is encoded by the *hTERT* gene and contains both a protein component and a ribonucleic acidcomponent.^2^ The *hTERT* gene is not expressed in normal postnatal somatic cells, but its expression is elevated in stem, germ, and cancer cells.^4^ The expression of *hTERT* in cancer cells allows their continued division, maintains telomere length, and inactivates apoptosis. Various mechanisms enhance the regulation of *hTERT* in tumors, including amplifications, structural changes, mutations, and epigenetic changes.^5^ Epigenetic changes are DNA modifications, such as methylation, that do not alter the base sequence but may affect DNA expression. Methylation involves the addition of a methyl group to the cytosine base of cytosine-guanine dinucleotides.^5^

Circulating cell-free DNA (cfDNA) is single or double-stranded extracellular DNA that is released into the bloodstream as a result of cell apoptosis, necrosis, and secretion.^6^ Increased levels of cfDNA and epigenetic changes are commonly observed in cancer patients and are associated with the progression of the disease and response to treatment.^7,8^ In cancer patients, cfDNA contains sequences from tumors which can serve as biomarkers for early-stage detection or for guiding therapy.^9,10^ A better understanding of cfDNA properties, in particular the methylation status, could guide future protocols for monitoring tumor composition and stage.^11^ In addition, cfDNA analysis allows cancer screening through liquid biopsy,^9,6^ which has multiple advantages over tissue biopsies, such as not being limited to a single observational region, less invasiveness, and lower cost.^12^

## OBJECTIVE

This study aimed to investigate the methylation status of the *hTERT* gene in the tumors, surrounding tissues, and cfDNA of patients with breast cancer. Furthermore, the methylation status of *hTERT* in the cfDNA from blood collected on the day of mastectomy was compared with that from blood collected approximately one year later. To the best of our knowledge, this is the first study to track *hTERT* promoter methylation in cfDNA obtained at two different time points after breast cancer treatment.

## METHODS

### Study design and patient recruitment

This prospective study included 15 women aged 44 to 78 years (mean age 56.7 ± 9.6 years) diagnosed with breast carcinoma. Patients were admitted to the Institute of Gynecology, Universidade Federal do Rio de Janeiro, Brazil. All women underwent a total mastectomy as part of their treatment. Before surgery, patients were invited to voluntarily participate in the study, and those who agreed to participate signed a consent form after receiving all further clarifications. Recruitment took place between October 2018 and July 2021.The sample size was determined based on the availability of patients who met the study criteria during the recruitment period.

### Data collection and ethical approval

Demographic and clinical data were obtained from the patients’ medical records. This study was approved by the Clinical Research Ethics Committee of Hospital Universitário Clementino Fraga Filho, Universidade Federal do Rio de Janeiro, Brazil. Certificate: (CAAE) # 91406118.6.0000.5257, August 29, 2018.

### Material collection

During mastectomy, sections measuring approximately 1 cm^3^ were collected from the tumor and surrounding tissue of each patient for DNA analysis. In addition, 5 ml of peripheral blood was collected to analyze cfDNA. Another blood collection was made approximately one year later for the second cfDNA analysis. Sample collection and histopathological examination were performed at the Institute of Gynecology. Molecular analyses were performed at the Laboratório de Patologia Molecular, Hospital Universitário Clementino Fraga Filho.

### Circulating free DNA extraction

DNA was extracted from fresh blood serum using a Quick-gDNA MiniPrep kit (Zymo Research, Orange County, United States), nº D3024, according to the manufacturer’s standard protocol.

### Extraction of DNA from the tumor and surrounding tissue

DNA extraction from the tumor and surrounding tissue was performed as previously described by McCormick et al.,^13^ using the Ultra Pure™ Phenol: Chloroform: Isoamyl Alcohol kit (Invitrogen™, Carlsbad, United States) Cat. No. 15593-031.

### Methylation mechanism

DNA samples were modified with sodium bisulfite and analyzed using the Methylation-Specific Polymerase Chain Reaction method. DNA modification was performed using an EZ DNA Methylation-Gold^TM^ Kit (Cat. No: D5005 Zymo Research, Orange County, United States), according to the standard protocol established by the manufacturer.

### Polymerase chain reaction (PCR)

To confirm the integrity of the DNA extracted from the samples, a fragment of exon 5 of the *p53* gene was amplified by polymerase chain reaction (PCR). The amplification reaction was performed according to Pestaner et al.,^14^ generating a 274-base pair product. For the *hTERT* amplification, two pairs of primers were used as follows: *hTERT-U*(unmethylated) forward, 5′-GAGGTATTTCGGGAGGTTTCGC-3′ and *hTERT-U*reverse, 5′-ACTCCGAACACCACGAATACCG-3′ producing a fragment of 126 base pairs,^15^ and *hTERT-M* (methylated) forward, 5′-GGAGGTATTTTGGGAGGTTTTGT-3′and *hTERT-M* reverse, 5′CAAACTCCAAACACCACAAATACCA-3′ producing a fragment of 121 base pairs.^15^ PCR conditions were: initial denaturation at 96°C for 7 min followed by 35 cycles of 95°C for 1 min, 62°C for 1 min, and 72°C for 1 min. The final extension step was performed at 72°C for 5 min.

### Gel electrophoresis and staining

PCR products were electrophoresed on 10% polyacrylamide gels. along with a negative control and a DNA marker. Gels were stained by the silver nitrate method.^16^ Briefly, the DNA-containing gels were first fixed with ethanol and acetic acid, followed by impregnation with silver nitrate, and finally, incubation in sodium hydroxide and formaldehyde to reveal the DNA bands.

## RESULTS

**Figure 1:**
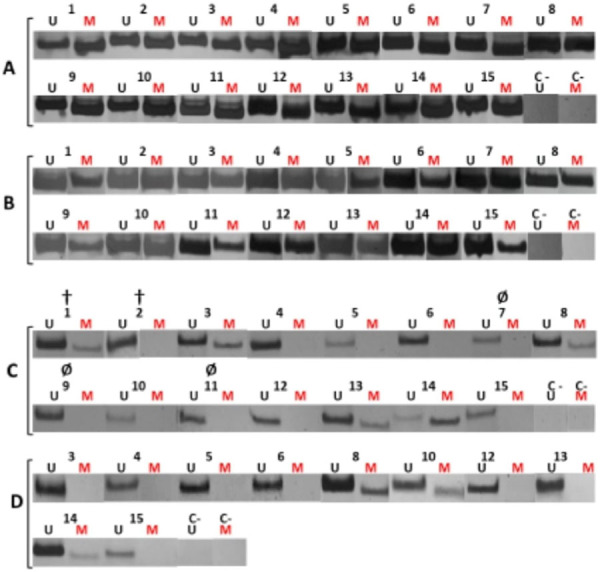
Methylation of the human telomerase reverse transcriptase (*hTERT*) gene. Numbers correspond to patients. A) tumor; B) tumor surrounding tissue; C) blood of the first collection; D) blood of the second collection.

Methylation of the *hTERT* gene was detected in all 15 patients in tumor cells and surrounding tissues (Figures 1A and B). Five patients (1, 3, 8, 13, and 14) displayed *hTERT* methylation in cfDNA from the blood of the first collection (Figure 1C). Two patients died after the first blood collection and three failed to return for the second blood collection for other reasons. Of the ten patients who returned for the second blood collection, three (8, 10, and 14) displayed *hTERT* methylation (Figure 1D). Methylation of *hTERT* was measured in patients 3 and 13in the first collection, but not in the second collection. Patient14 displayed less methylation in the second collection than in the first.

**Figure 2:**
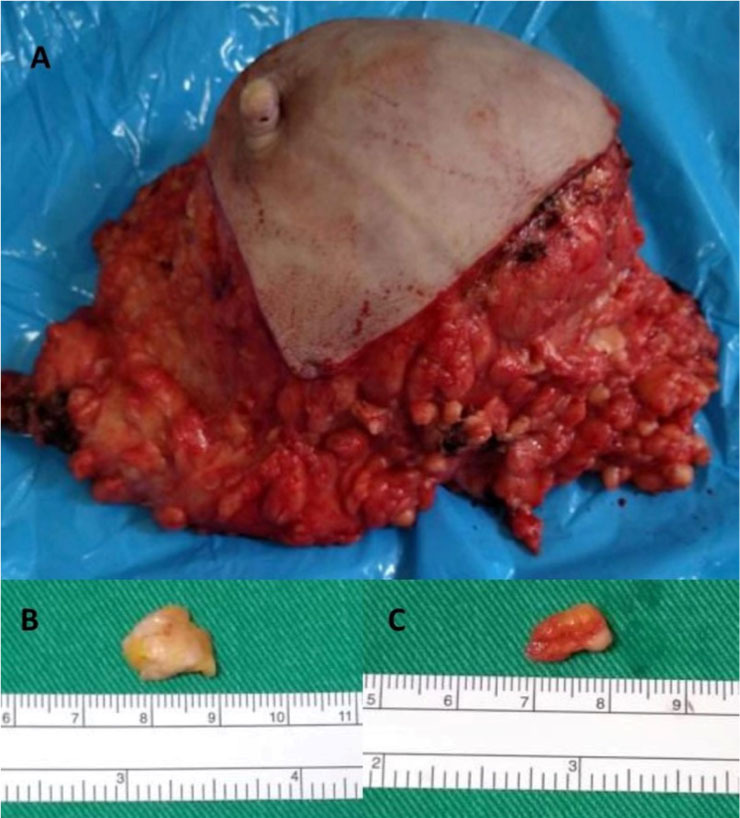
A) total breast section; B) tumor fragment; C) fragment of tumor-surrounding tissue. The top of the ruler is graduated in centimeters.

Figure 2 shows the total breast section removed from a patient (**A**), the tumor fragment (**B**), and the fragment of the surrounding tissue (**C**).

Table 1 shows the methylation panel of the *hTERT* gene in tumors, surrounding tissues, and blood cfDNA from the first and second collections.

**Table 1:** Panel of the *hTERT* gene methylation status

Patient	Tumor	Tumor-surrounding tissue	cfDNA of blood of first collection	cfDNA of blood of second collection
1	M	M	M	Death
2	M	M	U	Death
3	M	M	M	U
4	M	M	U	U
5	M	M	U	U
6	M	M	U	U
7	M	M	U	NR
8	M	M	M	M
9	M	M	U	NR
10	M	M	U	M
11	M	M	U	NR
12	M	M	U	U
13	M	M	M	U
14	M	M	M	M
15	M	M	U	U

*hTERT* = human telomerase reverse transcriptase; M = presence of methylated *hTERT*; U = no presence of methylated *hTERT*; NR = no return for second blood collection for reasons other than death; cfDNA = circulating free DNA.

Table 2 shows the patients’ age and clinical data (type of treatment and tumor stage).

**Table 2:** Age and clinical data of patients

Patient	Age	Treatment	TNM stage
1	53	NC	T4b N2 Mx
2	73	WD	WD
3	50	NC	WD
4	44	NC	T3 N0 M0
5	49	NC	CT3 CN2 CM0
6	59	NC	T4 N0 Mx
7	44	NC	T4B N1 Mx
8	49	NC	T3 N1 Mx
9	78	WD	WD
10	57	No treatment	T4 N0 Mx
11	57	NC	T4 N1 Mx
12	56	NC	T4B N0
13	59	Chemotherapy	T3 N1 M0
14	54	No treatment	T4b N2 Mx
15	69	NC	T3 N0 M0

TNM = tumor, node, metastasis; NC = neoadjuvant chemotherapy; WD = TNM stage not described in the records.

## DISCUSSION

The hTERT enzyme (encoded by the *hTERT* gene) plays a fundamental role in cancer formation by extending telomeres, thereby allowing tumors to avoid cellular senescence and apoptosis.^17^ Activity of hTERT is apparent in many cancers, but its expression is not observed in normal somatic cells. In contrast to other cancer-related genes, methylation of the *hTERT* promoter region does not lead to gene silencing, but instead has an upregulatory effect, increasing hTERT protein synthesis in tumor cells.^15^ For example, Haraguchi et al showed that hypermethylation of the *hTERT* gene in oral squamous cell carcinoma positively regulated hTERT enzyme synthesis, which was confirmed by immunohistochemistry.^15^

According to the scientific literature, changes in DNA methylation are an early event in tumorigenesis.^10^ For instance; one study showed that changes in methylation were detected in plasma four years before clinical diagnosis.^18^ Our study investigated the *hTERT* gene methylation status in breast cancer patients. DNA was extracted from tumors, surrounding tissues, and peripheral blood (cfDNA). The analyses revealed *hTERT* hypermethylation in tumors and surrounding tissues of all patients. This is very similar to the results reported by Masood et al.,^19^ who found that 94% of breast carcinomas displayed hypermethylation levels at least 2-fold higher than those measured in normal breast tissue. In contrast, in our study, only five of the fifteen patients presented *hTERT* methylation in their cfDNA. This may be due to the efficacy of neoadjuvant chemotherapy before mastectomy; a successful treatment tends to considerably diminish the release of tumor cfDNA into the bloodstream. Furthermore, two patients (3 and 13) displayed methylation in the first blood collection but not in the second, which may also be indicative of the success of treatment before surgery. Similarly, patient 14 showed less methylation in the second collection than in the first, suggesting the effectiveness of neoadjuvant chemotherapy in reducing tumor cfDNA release into the bloodstream.

## CONCLUSION

Our finding that only one-third of patients displayed methylation in their circulating DNA may be related to the success of chemotherapy. These results suggest that analysis of *hTERT* methylation in cfDNA could be useful in the follow-up of patients with breast cancer and in the evaluation of their response to treatment. Further studies are required to analyze the methylation of other cancer-related genes in the cfDNA of patients with breast cancer.
